# Management of treatment-resistant depression in primary care: a mixed-methods study

**DOI:** 10.3399/bjgp18X699053

**Published:** 2018-09-25

**Authors:** Nicola Wiles, Abigail Taylor, Nicholas Turner, Maria Barnes, John Campbell, Glyn Lewis, Jill Morrison, Tim J Peters, Laura Thomas, Katrina Turner, David Kessler

**Affiliations:** Centre for Academic Mental Health, Population Health Sciences, Bristol Medical School, University of Bristol, Bristol.; Centre for Academic Mental Health, Population Health Sciences, Bristol Medical School, University of Bristol, Bristol.; Centre for Academic Mental Health, Population Health Sciences, Bristol Medical School, University of Bristol, Bristol.; Centre for Academic Mental Health, Population Health Sciences, Bristol Medical School, University of Bristol, Bristol.; Primary Care Research Group, University of Exeter Medical School, Exeter.; Division of Psychiatry, Faculty of Brain Sciences, University College London, London.; Institute of Health and Wellbeing, General Practice and Primary Care Group, University of Glasgow, Glasgow.; Bristol Medical School, University of Bristol, Learning and Research, Southmead Hospital, Bristol.; Centre for Academic Mental Health, Population Health Sciences, Bristol Medical School, University of Bristol, Bristol.; Centre for Academic Primary Care, Population Health Sciences, Bristol Medical School, Bristol; National Institute for Health Research Collaboration for Leadership in Applied Health Research and Care West (NIHR CLAHRC West), University Hospitals Bristol NHS Foundation Trust, Bristol.; Centre for Academic Mental Health, Population Health Sciences, Bristol Medical School, University of Bristol, Bristol.

**Keywords:** antidepressants, depression, management, mixed methods, primary care, treatment resistance

## Abstract

**Background:**

Non-response to antidepressant medication is common in primary care. Little is known about how GPs manage patients with depression that does not respond to medication.

**Aim:**

To describe usual care for primary care patients with treatment-resistant depression (TRD).

**Design and setting:**

Mixed-methods study using data from a UK primary care multicentre randomised controlled trial.

**Method:**

In total, 235 patients with TRD randomised to continue with usual GP care were followed up at 3-month intervals for a year. Self-report data were collected on antidepressant medication, number of GP visits, and other treatments received. In addition, 14 semi-structured face-to-face interviews were conducted with a purposive sample after the 6-month follow-up and analysed thematically.

**Results:**

Most patients continued on the same dose of a single antidepressant between baseline and 3 months (*n* = 147/186 at 3 months, 79% (95% confidence interval [CI] = 73 to 85%)). Figures were similar for later follow-ups (for example, 9–12 months: 72% (95% CI = 63 to 79%). Medication changes (increasing dose; switching to a different antidepressant; adding a second antidepressant) were uncommon. Participants described usual care mainly as taking antidepressants, with consultations focused on other (physical) health concerns. Few accessed other treatments or were referred to secondary care.

**Conclusion:**

Usual care in patients with TRD mainly entailed taking antidepressants, and medication changes were uncommon. The high prevalence of physical and psychological comorbidity means that, when these patients consult, their depression may not be discussed. Strategies are needed to ensure the active management of this large group of patients whose depression does not respond to antidepressant medication.

## INTRODUCTION

Antidepressants are usually the first line of treatment for those with moderate or severe depression in primary care.^1^ In 2014, 57.1 million prescriptions for antidepressants were dispensed, more than double the number issued 10 years earlier (28.2 million in 2004).^2^ There is evidence to suggest that the increase in prescribing seen in recent years is largely the result of more patients receiving long-term treatment.^3^^–^5 In one fairly recent study, 47% of patients had been taking the same antidepressant for more than 2 years.^6^

However, many patients do not respond to antidepressants.^7^ In UK primary care, 55% of patients who have taken antidepressants for at least 6 weeks continue to have significant depressive symptoms.^8^ In the event of non-response (or inadequate response) to a course of antidepressants, National Institute for Health and Care Excellence (NICE) guidance suggests switching antidepressants (either within or between classes), augmenting medication with a drug that is not an antidepressant, or combining two antidepressants.^1^ Yet, there is little robust evidence for any of these strategies^1^ and little is known about how GPs manage patients whose depression does not respond to treatment with antidepressant medication.

The CoBalT study was a large multicentre randomised trial that investigated the effectiveness of cognitive behavioural therapy (CBT) as an adjunct to usual care for patients with treatment-resistant depression (TRD) compared with usual GP care.^9^^,^^10^ This paper uses quantitative and qualitative data collected during the CoBalT trial to describe usual care for primary care-based patients with TRD.

## METHOD

### The CoBalT trial

The CoBalT study was a randomised controlled trial examining the effectiveness of CBT as an adjunct to usual care that included pharmacotherapy for primary care patients with TRD.^9^^–^11 Between November 2008 and September 2010, participants were recruited from 73 general practices from three UK centres (Bristol, Exeter, and Glasgow). Eligible participants were: aged between 18–75 years; had been taking antidepressant medication at an adequate dose for at least 6 weeks; scored ≥14 on the Beck Depression Inventory (BDI-II) (2nd version);^12^ and met the ICD-10 criteria for depression (assessed using the Clinical Interview Schedule — Revised form.^13^ Assessment of adherence to antidepressants used the Morisky scale,^14^^,^^15^ with an additional item added to ensure that individuals who had missed fewer than two consecutive doses were not excluded. GPs excluded patients who had: bipolar disorder, psychosis, or major alcohol and/or substance misuse problems; were unable to complete the questionnaires; and were pregnant. Individuals who were receiving CBT or other psychotherapy or secondary care for their depression, who had received CBT in the past 3 years, or who were taking part in another intervention study were also excluded.

**How this fits in**

Non-response to antidepressant medication is a substantial problem in primary care but little is known about how GPs manage patients with depression that does not respond to such treatment. Using data collected as part of the CoBalT multicentre randomised controlled trial, it was found that changes to antidepressant medication are uncommon in primary care patients with treatment-resistant depression. Many just collect repeat prescriptions for their medication, and the high prevalence of physical and psychological comorbidity means that their depression may not be discussed during contact with their GP. Therefore, it is important that strategies are put in place for the active management of the large number of patients whose depression does not respond to an initial course of antidepressant treatment.

Participants were randomised into one of two groups: 1) to continue with usual care from their GP; or 2) to receive the intervention (12–18 sessions of individual face-to-face CBT) in addition to usual care. The study found that CBT, given in addition to usual care, was effective in reducing depressive symptoms and improving quality of life in patients with TRD, compared with usual care alone.^10^ This paper focuses on the data collected for those individuals randomised to continue with usual care from their GP.

### Quantitative data

Participants were followed up at 3-month intervals for a year. At each follow-up, participants were asked whether they were still taking antidepressant medication and, if so, to provide details (name and dose) of the antidepressant medication(s).^9^ On two occasions, participants were also asked about the number of visits to their GP (between baseline and 6-month follow-up; and between 6- and 12-month follow-ups). In addition, data on other treatments received (including computerised/online CBT; counselling or other ‘talking therapies’; and use of secondary care) were also recorded at the 6- and 12-month follow-ups.

### Qualitative interviews

A qualitative study was integrated within the CoBalT trial.^11^ One of its primary objectives was to explore trial participants’ experiences of usual GP care before and during the trial. Patients were invited to take part in an interview after completion of their primary outcome measures for the trial at 6 months post-randomisation. Consent to be approached for interview had been secured during their baseline assessment for the trial.

A purposeful sampling strategy was used to ensure interviews were held with participants who varied in relation to the trial arm, study site (Bristol, Exeter, and Glasgow), age, sex, socioeconomic background (based on educational attainment, employment and housing tenure), and whether they met criteria for ‘response’ (the primary outcome, defined as at least a 50% reduction in depressive symptoms on the BDI-II at 6 months compared with baseline).

Face-to-face interviews took place at the participant’s home or their GP surgery, as preferred. A topic guide was used to ensure consistency across the interviews. Participants were asked about their experiences of depression, what treatments and support they had received before and during the study, and, specifically, their views and use of antidepressants.

### Data analysis

#### Questionnaire data

The quantitative data were analysed using Stata version 13.1. At baseline, 3-, 6-, 9-, and 12-month follow-up, the number of individuals who were taking different types of antidepressants (or combination therapy) were tabulated. Comparisons between data collected at: baseline and 3 months; 3 and 6 months; 6 and 9 months; and 9 and 12 months were undertaken in order to determine the number of participants who:

- had continued on the same medication;
- had an increased dose of medication;
- had switched antidepressant medication;
- received a second antidepressant (combination therapy) or whose treatment was augmented with another drug; or
- stopped (or re-started) antidepressants.

Analyses were repeated for the subset of participants who reported taking antidepressants throughout the 12-month follow-up. Descriptive statistics were used to summarise the number of GP visits and other treatments received during the 12 month follow-up (in terms of computerised CBT, counselling or ‘talking therapy’, CBT and treatment in secondary care).

#### Interview data

Participants gave consent for their interview to be audiorecorded and transcribed verbatim. Data collection and analysis took place in parallel, so that earlier data collection could inform the focus of later interviews, and to enable the researchers to establish when data saturation had been reached, that is, no new themes emerged from the analysis.

The data were analysed thematically,^16^ as this permitted comparisons to be made within, and across, the interviews. This involved team members reading and re-reading transcripts in order to familiarise themselves with the data, identify emerging themes, and develop a coding frame. This coding frame was refined through team members independently coding a sample of transcripts and then discussing their interpretation and coding of the data. Once the coding frame had been finalised, transcripts were electronically coded using the software package ATLAS.ti. Data were then systematically analysed using an approach based on framework analysis.^17^ Using this method, participants’ views about specific issues were summarised in tables and comparisons then made between the accounts of participants of differing age, sex, and socioeconomic backgrounds.

## RESULTS

### Baseline characteristics of CoBalT participants and follow-up rates

In total, 235 patients were randomised to continue with usual care from their GP.^10^ The mean age was 50 years (SD 11.5 years) and most (*n* = 178, 76%) were female. At baseline, their mean BDI-II score was 31.8 (SD 10.9). Twenty-eight per cent of patients (*n* = 65) met ICD-10 criteria for a severe depressive episode and the duration of their current episode of depression was at least 2 years for 60% of patients (*n* = 140). Eighty-nine percent (*n* = 210) had a secondary diagnosis of an anxiety disorder based on the Clinical Interview Schedule — Revised version (CIS-R) and 77% (*n* = 181) self-reported a longstanding illness or disability.

The number followed up with data at baseline and 3 months; 3 and 6 months; 6 and 9 months; and 9 and 12 months was 219 (93%), 207 (88%), 191 (81%), and 181 (77%) respectively. The mean BDI-II scores at follow-up have been previously published.^10^ For those in the usual-care group included in the present analyses, the BDI-II scores at follow-up were: mean 24.4 (SD 13.1) at 6 months and mean 21.3 (SD 12.9) at 12 months.

### Self-reported data on use of antidepressant medication and other health care

At baseline, 214 (91%) of participants in the usual-care arm were taking a single antidepressant (monotherapy), with citalopram and fluoxetine being the two most common drugs (Table 1). Most patients (*n* = 152, 71%) had taken this medication for more than 12 months. Twenty-one individuals (9%) were taking two antidepressants (combination therapy), though in 12 cases this included low-dose (<75 mg) amitriptyline (Table 1). Most participants continued to take antidepressants during the follow-up period (Table 2).

**Table 1.** Antidepressant medications at baseline

**Antidepressant**	**Dose, mg**	**Antidepressant**	**Dose, mg**	***n***	**%**
**Monotherapy (*n* = 214)**					
Citalopram	20–60			71	30.2
Fluoxetine	20–60			70	29.8
Venlafaxine	75–300			18	7.7
Mirtazapine	30–60			15	6.4
Paroxetine	20–40			12	5.1
Dosulepin	150–175			6	2.6
Sertraline	100–150			6	2.6
Lofepramine	140–210			5	2.1
Escitalopram	10–40			3	1.3
Amitriptyline	150–180			3	1.3
Duloxetine	60–90			2	0.9
Trazodone	150–300			2	0.9
Reboxetine	8			1	0.4
**Combination therapy (*n* = 21)**					
Fluoxetine	20–40	Amitriptyline	20–120	9	3.8
Citalopram	20–50	Amitriptyline	10–150	5	2.1
Citalopram	20–40	Mirtazapine	15–22.5	2	0.9
Citalopram	10	Venlafaxine	75	1	0.4
Fluoxetine	80	Citalopram	20	1	0.4
Paroxetine	40	Amitriptyline	20	1	0.4
Sertraline	100	Nortriptyline	100	1	0.4
Venlafaxine	150	Amitriptyline	225	1	0.4

**Table 2.** Changes in antidepressant medication taken during follow-up

		**Baseline–3 months (*n* = 219)**	**3–6 months (*n* = 207)**	**6–9 months (*n* = 191)**	**9–12 months (*n* = 181)**
**No longer taking antidepressants (already stopped + new stops)**		8	14	11	14

**Still taking antidepressants**		211	191	175	165

**Re-started taking antidepressants**		0	2	5	2

**1st time point**	**2nd time point**	**Baseline–3 months (*n* = 203)^a^**	**3–6 months (*n* = 181)^a^**	**6–9 months (*n* = 170)^a^**	**9–12 months (*n* = 161)^a^**

**Monotherapy**	**Continued on same drug**	**176**	**156**	**142**	**131**
	Same dose	147	130	113	103
	Increased dose	17	14	15	17
	Reduced dose	12	12	14	11

	**Switched antidepressant**	**5**	**2**	**3**	**6**
	Within class	1	1	1	2
	Between class	4	1	2	4

	**Combination therapy/augmented antidepressant**	**5**	**5**	**4**	**7**
	Second antidepressant^b^	5	5	4	7
	Non-antidepressant augmenter	0	0	0	0

**Combination therapy**	**Continued on combination therapy^b^**	**14**	**15**	**15**	**14**
	Same combination of drugs^c^	14	15	14	13
	Different combination	0	0	1	1

	**Change to monotherapy**	**3**	**3**	**6**	**3**

**Missing information on type and/or dose of antidepressant**		8	10	5	4

a Number of patients with data on type and dose of antidepressant medication among those still taking antidepressants.

b *Includes combinations where the second antidepressant is low-dose (*<*75 mg) amitriptyline.*

c Dose of medication may have changed.

Of the 186 patients who were taking a single antidepressant at baseline and who were followed up at 3 months, the majority (*n* = 147, 79%) continued on the same dose of medication at 3 months (Table 2, total number on monotherapy = number continued on same drug, switched antidepressant and changed to combination/augmented treatment). Figures were similar for the later follow-ups (130 out of 163 (80%) at 6 months; 113 out of 149 (76%) at 9 months; and 103 out of 144 (72%) at 12 months). Of those who continued on the same antidepressant, only a small percentage (9–13%) were taking an increased dose of their antidepressant at the subsequent follow-up (Table 2). Switches to a different antidepressant or the addition of a second antidepressant treatment were uncommon (Table 2). Most patients who were taking two antidepressants at baseline continued with combination therapy during follow-up (Table 2).

In the subset of 145 patients who completed all the follow-ups and who reported taking antidepressants at all time points, 116 (80%) reported taking the same medication(s) throughout and 69 (48%) reported no change in the dose of their medication(s) (further details on the subset who took antidepressants throughout follow-up are available from the authors on request).

The percentage who switched to a different antidepressant or who were taking two antidepressants were similar to the figures using all available data.

On average, patients visited their GP four times between baseline and the 6-month follow-up (*n* = 210; mean 4.1 [SD 3.5]) and on a similar number of occasions between 6 and 12 months (*n* = 197; mean 4.1 [SD 4.9]). Few patients reported using any computerised CBT packages during the 12-month follow-up (Table 3).

**Table 3.** Other treatments received during follow-up among those in the usual-care group

	**6-month follow-up**			**12-month follow-up**		
**Other treatment received**	**N**	***n***	**%**	**N**	***n***	**%**
Used any CBT packages during last 6 months	213	7	3.3	198	3	1.5
Had any counselling or ‘talking therapy’ during the last 6 months	213	23	10.8	197	33	16.8
Had CBT during the last 6 months	213	6	2.8	197	12	6.1
0–5 sessions of CBT	213	2	0.9	197	3	1.5
6–8 sessions of CBT	213	2	0.9	197	5	2.5
≥9 sessions of CBT	213	2	0.9	197	4	2.0
**Use of hospital or secondary care services because of mental health problems**						
NHS hospital for an overnight stay	211	0	0	196	3	1.5
Visited Emergency Department for mental health problems	211	3	1.4	196	3	1.5
NHS outpatient or community mental health team clinics	211	3	1.4	196	12	6.1
Private hospital or clinics for mental health problems	211	0	0	196	1	0.5

CBT = cognitive behavioural therapy.

Less than 25% of participants had received counselling or ‘talking therapy’ during the 12-month follow-up and the number receiving CBT was very small (Table 3: *n* = 4 had ≥9 sessions of CBT at 12-month follow-up).

Few participants had received outpatient treatment at an NHS hospital or community mental health team clinic (Table 3).

### Characteristics of participants who took part in the face-to-face interviews

Interviews with the 14 usual-care participants lasted, on average, 51 minutes (range: 24–85 minutes). The characteristics of those interviewed were similar to those who participated in the CoBalT trial generally^10^ (Table 4). Mean age was 45 years, the majority (71%, *n* = 10) were female, and 57% (*n* = 8) were home owners. All had a secondary diagnosis of an anxiety disorder.

**Table 4.** Baseline sociodemographic and clinical characteristics of participants interviewed (*N* = 14)

**Baseline characteristic**		
Age, years, mean (SD)	45	(11.8)

Female, *n* (%)	10	(71.4)

Educational background: *n* (%)		
GCSE/no formal qualifications	6	(42.9)
A Levels/higher diploma/degree	8	(57.1)

Employment status: *n* (%)		
Paid employment (full/part-time)	6	(42.9)
Not in employment	5	(35.7)
Not employed due to ill health	3	(21.4)

Housing: *n* (%)		
Homeowner	8	(57.1)
Tenant/living with relative/other	6	(42.9)

Baseline BDI-II score, mean (SD)	32.6	(7.4)

Duration of depression,>2 years: *n* (%)	6	(42.9)

Been on current antidepressants for>12 months: *n* (%)	11	(78.6)

Secondary diagnosis of anxiety disorder: *n* (%)	14	(100.0)

BDI-II score at 6-month follow-up, mean (SD)	23.2	(9.5)

SD = standard deviation.

### Interview findings

Findings have been presented below under headings that relate to the main areas explored with participants in relation to usual care. They also reflect the main areas discussed above during the description of the quantitative findings, to aid comparisons between the two sets of findings presented in this paper.

#### Participants’ views and experiences of antidepressants

Participants’ accounts of usual care for their depression described how this had mainly entailed taking antidepressants. Within the accounts there were themes around medication helping to manage rather than cure depression, a lack of change of antidepressant and active monitoring once on medication, and a fear of stigmatisation and a perceived dependency as a result of taking an antidepressant.

The role of medication was described as maintaining a ‘baseline’ level of functioning to prevent further deterioration in mood, rather than addressing the underlying problem:

*‘I think they help me, they sort of give me a sort of baseline to work from … it’s* [the antidepressant] *trying to control this chemical imbalance or something I’ve got in my brain and it sort of helps sort of keep it on a level, as if I’m not going to drop too far down … I don’t know quite how to explain it, but it makes me feel safe.’*

(Female, aged 53 years, Bristol)

Many participants commented that they had been taking antidepressants for years, often trying multiple antidepressants and doses before settling on their current regimen. Some patients reported taking antidepressant medications primarily for indications other than depression, such as anxiety disorders and neuropathic pain. All the participants reported continuing to take the same antidepressant during the trial but several people spoke about altering the dose of their medication if they felt their mood was getting better or worse.

Almost all the participants mentioned not wanting to continue antidepressants long term. Reasons included a fear of taking something *‘unnatural’*, anxiety about missing doses, and feeling one *‘ought’* to be able to cope without them. However, patients were reluctant to reduce or stop their medication, even when their mood had improved.

Participants were concerned that their symptoms would worsen if they stopped medication, and several participants described feeling unwell when they missed doses or abruptly discontinued medication, which they interpreted as evidence of ongoing depression:

*‘The thing is you only know with these things* [antidepressants] *that they’re doing some sort of good when you try to give them up and then suddenly you feel twice as bad.’*

(Male, aged 60 years, Exeter)

‘I tried to come off medication months ago and I had a couple of little wobbles and stuff so I went back on it.’

(Female, aged 26 years, Exeter)

Some participants also explained that they were waiting for a less stressful time in life, such as retirement, before stopping medication. Several participants described continuing antidepressants simply because their GP continued to issue prescriptions and had not discussed stopping medication with them. Some seemed reluctant to raise the topic of medication reviews during consultations, either because they felt it was not their role or because they came to appointments with multiple medical issues that were prioritised over antidepressant reviews:

‘The only reason I still take them now is because a) I haven’t actually technically been told not, you know to come off them, and b) I just think it’s not a good idea to just suddenly stop them like I did last time.’

(Female, aged 26 years, Exeter)

‘I’ll come with my list of things and get them done but I would forget unless, I kind of … should the GP be doing that, saying “let’s have a look at the depressives, let’s see what they’ve been on and how long” and then bring them in and let’s have a go here, and to see whether they’re still depressed or whatever or whether it is the medication that’s causing it.’

(Male, aged 60 years, Glasgow)

#### Participant’s experiences of GP care

The participants’ accounts suggested the care they received from their GP had not changed since they entered the trial. All but two participants reported no change in how often they saw their GP during the study period, and the two individuals who said they were attending more frequently did so to collect sick notes or to monitor their weight. Most visits related to physical health, particularly musculoskeletal problems, rather than mood. Antidepressants were generally collected as repeat prescriptions and most participants did not describe active review by the GP:

Interviewer: ‘*So what care has your GP given you? Do you just see them when you need more antidepressants or do you …’*

Patient: ‘*I don’t necessarily see them about that. Just get a repeat prescription.’* (Female, aged 41 years, Bristol)

*‘* [I see the GP] *once a month only because I’m on the diet and he’s weighing me once a month. Otherwise it would be once whenever I needed a repeat prescription … which is normally three monthly.’*

(Female, aged 39 years, Exeter)

Across the accounts there were themes around a lack of time, continuity, and expertise. Many patients felt that their GP did not have enough time to spend with them and described a lack of continuity in terms of which GP they saw. Some expressed a belief that because GPs are ‘generalists’ they do not have adequate understanding of depression:

‘It’s as quick as they can get you out, write a script and out you go again.’

(Female, aged 51 years, Glasgow)

‘I think GP support is helpful but it is very limited and I don’t think they really know enough about depression either. Or as much as perhaps they ought to considering that so many people seem to have depressive symptoms.’

(Female, aged 29 years, Bristol)

However, there were exceptions with two participants stating that their GP closely monitored their mood and offered frequent medication reviews and referrals, though it was apparent that even patients who felt closely monitored remained on a stable dose of medication during the trial:

‘She’s been keeping quite a close eye on how I am and listening to me, she’s very good like that … Each time I see her she says well come back and see me such and such and we’ll see how you’re going then and we’ll discuss again if you want to come down off the tablets.’

(Female, aged 53 years, Bristol)

#### Experiences of seeking additional care

Very few participants said that they had accessed secondary care services or psychotherapy during the trial. Some participants cited barriers to accessing therapy, such as lack of local services, long waiting lists, and cost (for treatments not available on the NHS). Other participants mentioned that attending appointments would be difficult due to a lack of transport, because they had care or work commitments, or were anxious about leaving the house. Some participants seemed to be struggling with motivation, waiting for someone else to suggest therapy, or believing that it would not work for them. Others were dissuaded from seeking further treatment, having previously experienced counselling or therapy as unhelpful:

‘I’d got absolutely nowhere, because I mean I wasn’t understanding what he was getting at and that would make the whole thing utterly futile … it was difficult and I could not relate what happened during the day to a depressive episode.’

(Male, aged 60 years, Glasgow)

Other participants mentioned that they did not feel the need for psychological treatment because their symptoms had improved or they did not feel ‘depressed’. In addition, many participants described having developed their own coping strategies. These included accessing complementary therapies and support from family and friends.

## DISCUSSION

### Summary

Most patients whose depression had not responded to antidepressants were taking a single antidepressant and, over a period of 12 months, most continued to take the same medication, often at the same dose. Switching to a different antidepressant or starting a second antidepressant (combination therapy) was uncommon. Patients reported visiting their GP, on average, eight times over the 12-month follow-up period. However, the interview data suggested that these consultations were often for other health problems and many patients reported getting their antidepressants via repeat prescriptions. Use of computerised CBT packages was infrequent. Less than 20% of patients had received some form of counselling or ‘talking therapy’ over the 12 months, with very few receiving a course of CBT or having been referred to secondary care.

Patients recruited to the CoBalT trial had severe and chronic depression, often with physical and psychological comorbidity. Many patients spoke about the fact that they had been on antidepressants for years and during that time had tried a number of different antidepressants. Changes in the type of medication were sometimes made in response to side effects but not all patients were clear about the rationale for changing or re-starting a different type of medication. Many patients clearly saw their depression as a chronic illness — increasing or decreasing the dose of their medication in response to a worsening or improvement in their depression. Antidepressants were not seen as the solution by this group but rather a way of helping them to manage their symptoms. Some patients were unsure as to whether the medication was helpful and many expressed a desire to stop taking antidepressants and wanted help to do this.

### Strengths and limitations

CoBalT was a large multicentre randomised trial based in 73 general practices in UK primary care. An inclusive definition of treatment resistance was used, hence patients recruited to CoBalT were likely to be representative of those whose depression has not responded to treatment with antidepressants in primary care. The authors used self-reported data on antidepressant use that was collected on multiple occasions during the 12-month follow-up. As such, there were data on what patients were taking, rather than making assumptions about medication usage based on prescription data. Follow-up rates over the 12 months were high, with patterns of antidepressant medication use similar for those who provided data at all time points compared with those who provided data only at some time points.

It is important to acknowledge that participants in this study were taking part in a randomised trial. Therefore, as GPs were aware that these individuals had been randomised to continue with usual care (rather than receive the intervention), it was possible that GPs could have been more proactive in their management of this patient group (performance bias). However, there was little evidence of this either in terms of the number of GP appointments, antidepressant medication taken, or use of secondary care when compared with those randomised to usual care with the intervention group.^18^ Furthermore, accounts of usual care were similar for those who received CBT.^11^ This was a group of patients with severe and chronic depression. The majority of participants had been taking antidepressant medication for more than 12 months. The authors did not have data on their depression scores when antidepressant medication was first prescribed. Participants may, therefore, have experienced partial response to treatment, and changes to their medication may have taken place before entry to the trial. Qualitative data indicate that the latter had happened in some cases.

## Comparison with existing literature

To the best of the authors’ knowledge, there are no previous studies describing usual care for primary care-based patients whose depression has not responded to antidepressant medication. The lack of focus on this group in primary care may be, in part, because the term ‘treatment-resistant depression’ is not commonly used in primary care and may be thought to describe those in secondary care, rather than referring to those whose depression does not respond, or only partially responds, to treatment with medication. Although the earliest definition of ‘treatment resistance’ was based on non-response to at least 4 weeks of antidepressant medication,^19^ later definitions have focused on non-response to multiple courses of antidepressants.^20^ Yet, as the authors have shown in a previous paper,^8^ there are many patients in primary care who continue to have significant depressive symptoms following treatment with antidepressants, and this inadequate response to treatment is an important public health problem.

There is good evidence for the clinical and cost-effectiveness of individual ‘high intensity’ CBT^10^^,^^21^ over the long-term but it is clearly important that novel interventions are evaluated for the treatment of the large number of patients whose depression does not respond to treatment with antidepressant medication. More active management of such patients in primary care may improve outcomes for this group.

The 2017/2018 Quality and Outcomes Framework (QOF), which *‘rewards practices for the provision of quality care’*, has only a single item for depression (DEP003).^22^ As such, reviews of patients newly diagnosed with depression are commonplace but, though the current NICE guidelines for depression^1^ encourage review of those taking antidepressants, there is no requirement to record symptoms and no incentive for GPs to actively manage depression in the longer term. Indeed, other depression items in the QOF were dropped in recent years, which may have contributed to the lack of active management. This contrasts with long-term physical health conditions such as diabetes, chronic obstructive pulmonary disease (COPD), and cardiovascular disease. As an example, the QOF requires practices to maintain a register of those with diabetes and COPD and has, respectively, nine and three indicators related to ongoing management. As a consequence, the care of long-term physical health conditions is much more structured and openly discussed.

A meta-analysis of the effectiveness of disease management programmes provides support for such programmes for depression,^23^ though a collaborative care model for depression evaluated in the UK had only modest benefits^24^ and others have highlighted a lack of confidence among some GPs in managing patients with depression compared with those with long-term physical conditions.^25^ A recent report from the King’s Fund^26^ also highlights, in line with the authors’ data, the lack of follow-up for patients with depression. The latter may be one of the most important elements of high-quality care for patients with depression.^27^ The latest revisions of the NICE depression guidelines, currently in draft (https://www.nice.org.uk/guidance/indevelopment/gid-cgwave0725/documents), include recommendations to review how well treatments are working and thus may encourage more active follow-up.

### Implications for practice

The NHS mandate^28^ requires NHS England to ensure ‘parity of esteem’ between mental and physical health conditions, and to make improvements in the way that long-term conditions are managed. It is standard practice in primary care to have nurse-led clinics to review the care of those with long-term physical conditions such as diabetes, COPD, and cardiovascular disease. It is time to ensure that equivalent strategies are in place for the active management of the large number of patients whose depression does not improve after an initial course of antidepressant treatment.
